# The impact of software updates on accuracy of intraoral scanners

**DOI:** 10.1186/s12903-023-02926-y

**Published:** 2023-04-15

**Authors:** Judit Schmalzl, Ivett Róth, Judit Borbély, Péter Hermann, Bálint Vecsei

**Affiliations:** Department of Prosthodontics, Semmelweis University, Szentkiralyi Street 47, 1088 Budapest, Hungary

**Keywords:** Accuracy, Trueness, Precision, Software update, Intraoral scanner

## Abstract

**Background:**

Digital workflow is showing an increasing tendency in everyday dentistry. Accuracy is essential during digital dental workflows for all indication areas. The present study aimed to evaluate the effect of software updates on the accuracy of intraoral scanner (IOS) devices.

**Methods:**

3Shape Trios 3 Pod with software versions 18.1.2. (TRI3_1) and 20.1.2. (TRI3_2); 3Shape Trios 4 Move, version 19.2.2. (TRI4_1); and 3Shape Trios 4 Pod, version 20.1.1. (TRI4_2) were used to take direct optical impressions from a polymethyl methacrylate (PMMA) full arch reference model with prepared teeth (FDI 11,14,17 for crowns and FDI 26 for onlay) and an edentulous region (between FDI 14 and 17). The scanners were used eight times; STL files were imported into Geomagic Control X for accuracy assessment by comparing them to a reference data set created by an industrial high-precision scanner (AICON SmartScan-3D C5). The average deviation of the surface points was calculated in three locations: across a full arch (Parameter 1), the region of a four-unit bridge (Parameter 2), and a single prepared abutment (Parameter 3).

**Results:**

In parameter 1 and 2, the newest model with the latest software (TRI4_2) reached the highest accuracy (31.06 ± 5.24 µm and 21.69 ± 7.50 µm). In parameter 3, an older generation scanner running legacy software produced the highest accuracy: TRI4_1, 11.75 ± 0.35 µm.

**Conclusion:**

Appropriate software updates can significantly increase the trueness and precision of intraoral scanner devices. With updated software, the older generation can match the accuracy level of latest equipment.

## Background

Digital workflow is showing an increasing tendency in everyday dentistry. The beginning of digital dentistry can be placed in the early’80 s [1]. From that point on, digitalization has constantly been present and evolving in the dental field. By now, intraoral scanners easily exceed the accuracy of conventional impressions up to 4–5-unit bridges. Therefore, more and more dental offices invest to intraoral scanners (IOSs) [2]. Accuracy is essential during digital dental workflows for all indication areas. According to ISO 5725, accuracy comprises precision and trueness [3]. Accuracy means the difference between the quantitative values obtained from the measurement and the actual spatial values of the measured object [4]. Precision is the difference between the repeated measurements on a given target, and trueness expresses how close a measurement’s results are to the actual values of the measured object [5]. Crowns’ and bridges’ marginal and internal fit is the most important criteria for clinically acceptable prosthetic restorations [6]. Clinically acceptable accuracy can be placed between 50 and 120 μm (or less) according to the literature [5–10]. Previous studies show that digital workflow is clinically acceptable for solo restorations and short-span bridges [11, 12]. The accuracy of digital impressions decreases as more teeth are scanned [13, 14]. Most intraoral scanners create the 3D virtual model by capturing 2D images and stitching them together with some overlaps. Stitching errors add up to a greater inaccuracy along the full arch [15]. The challenge areas of digital intraoral scanning include implant-supported restorations, full-house bridges and edentulous ridges [16, 17]. Based on the literature, the scanning strategy (the order in which teeth surfaces are to be digitalized) determines the accuracy of digital impressions [18–20]. Mennito et al. [21] established that the scanning path influences the accuracy when larger areas are scanned, such as full arch scans. Moreover, Ender et al. [22] found that while the scanning strategy does not affect the accuracy on short-span segments, it has an impact on full arch. Another important aspect is the IOS’s calibration, which can significantly impact accuracy [23]. An additional factor of essence is the lightning conditions, which also affect accuracy [24]. Furthermore, the proficiency of the person who is scanning and the patient’s individual characteristics (such as arch width, extent of mouth opening) can influence the quality of digital impressions [25, 26]. It is well-known that the digital impression-taking has a learning curve; the operator must practice intraoral scanning before confidence in everyday clinical application is achieved [27–29].

There is less information in literature about the impact of the hardware and software components on IOS performance [2, 30]. The manufacturer companies continuously develop new generations of intraoral scanners (new hardware and software versions) and software updates (same generations of IOSs with new software version). The updates aim to improve the IOS’s overall performance and capability to capture the intraoral situation more reliably, stably, and rapidly – making digitizing easier for the operator a more comfortable for the patient.

Numerous studies examined the accuracy of intraoral scanners, but only a few attempted to evaluate the effect of software updates on accuracy [2, 30]. Trios 3 and Trios 4 were evaluated in our study since the 3Shape Trios scanners proved to have the high accuracy in the literature, and therefore they are an often used IOS [31, 32].

The present study aimed to investigate the effect of software updates on trueness and precision in case of two different generations of intraoral scanners with four different software versions. The null hypothesis was that there is no association between the software version of the IOSs and the accuracy of digital impressions. The alternative hypothesis was that the newer software version of the IOSs generates a more accurate digital impression.

## Methods

**Fig. 1:**
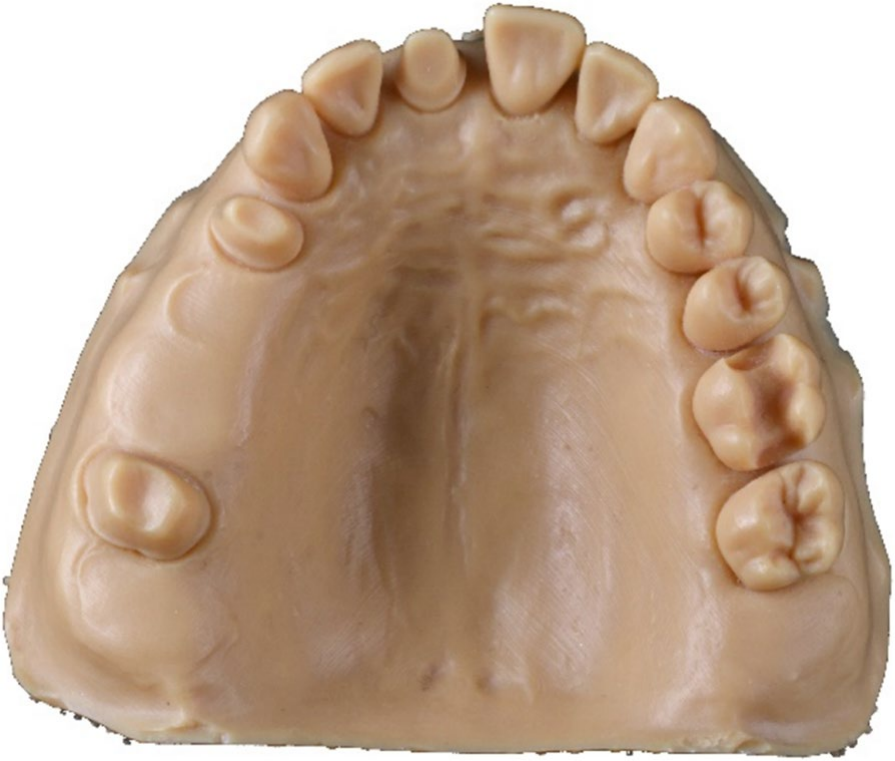
Reference polymethyl methacrylate model

Our study examined two generations of intraoral scanners with four different software versions (Table 1). The tested intraoral scanners included the 3Shape Trios 3 (2015) with the 18.1.2. software version introduced to the dental market in 2018 (TRI3_1) and with the 20.1.2. software version introduced in 2020 (TRI3_2), the 3Shape Trios 4 (2019) with the 19.2.2. software version presented in 2019 (TRI4_1), and the 3Shape Trios 4 (2019) with the 20.1.1. software version released to market in 2020 (TRI4_2). The investigated IOSs in the order of software release were the following: TRI3_1 (3Shape Trios 3 18.1.2.), TRI4_1 (3Shape Trios 4 19.2.2.), TRI4_2 (3Shape Trios 4 20.1.1.) and TRI3_2 (3Shape Trios 3 20.1.2.). Trios intraoral scanners use confocal laser scanning technology to capture the virtual model [29]. As a reference, a polymethyl methacrylate (PMMA) model was used (Fig. 1). Supragingival prepared teeth (FDI World Dental Federation) included numbers 11, 14, and 17 for a crown and 26 for an inlay; teeth 15 and 16 were missing.

**Table 1:** The scanners used in this study (generation and software version)

	**Generation**	**Sofwareversion**	**Release date**
TRI3_1	3Shape Trios 3 Pod®	18.1.2.	2018.
TRI3_2	3Shape Trios 3 Pod®	20.1.2.	2020.
TRI4_1	3Shape Trios 4 Move®	19.2.2.	2019.
TRI4_2	3Shape Trios 4 Pod®	20.1.1.	2020.

**Fig. 2:**
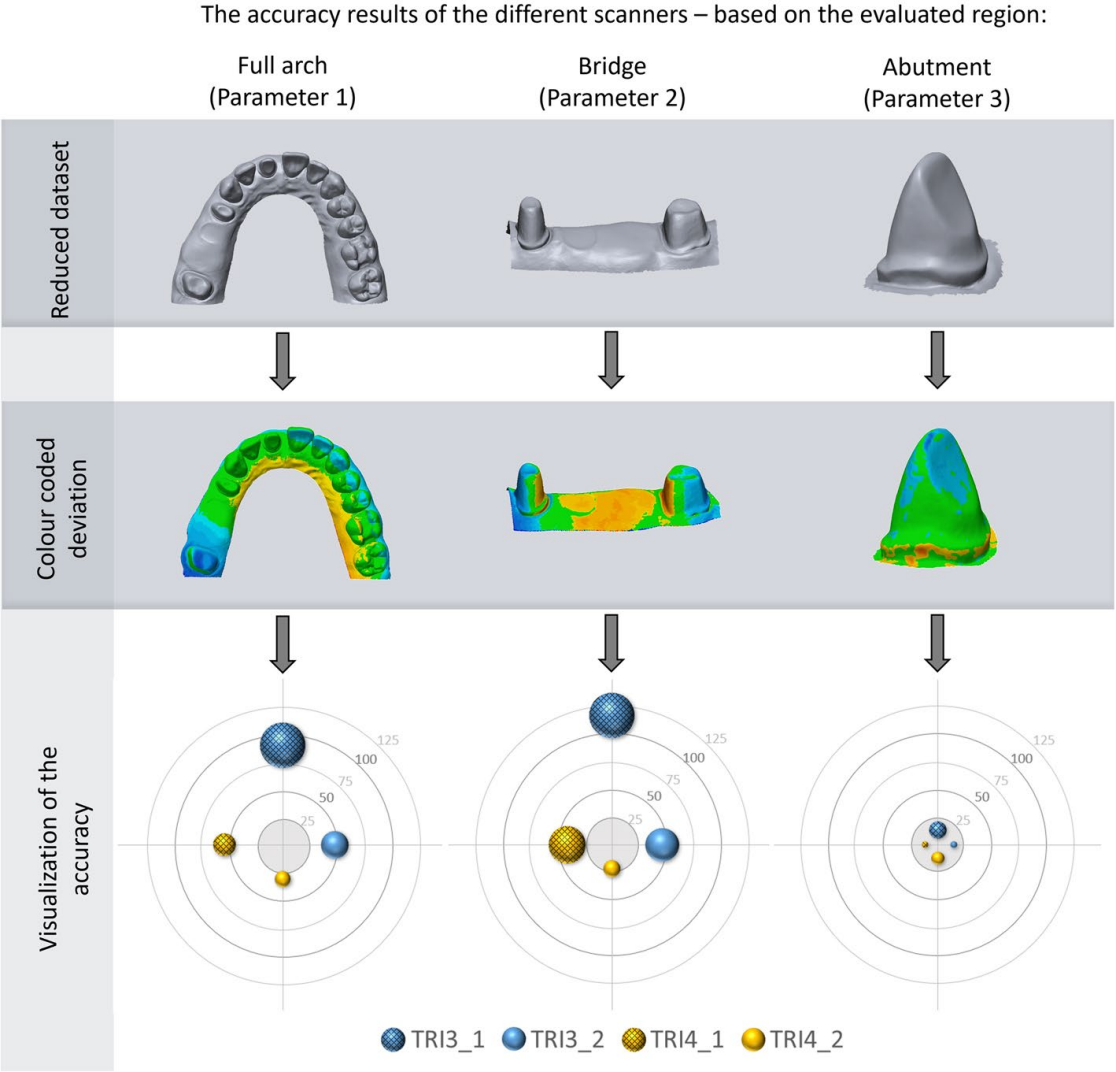
Measured parameters: full arch (Parameter 1), the region of a four-unit bridge (Parameter 2), and a single prepared abutment (Parameter 3)

The reference model was scanned by a highly accurate industrial scanner (AICON SmartScan-3D C5; AICON 3D Systems GmbH, Braunschweig, Germany). According to the user guide, the reference scanner’s accuracy was a maximal 8-μm feature accuracy (and a 2-μm resolution limit) [33]. An experienced operator familiar with Trios IOSs made all the digital impressions. The manufacturer-recommended scanning strategy was followed during the process. Scanning was started from the occlusal surface of the left prepared first molar. After capturing the occlusal surface, the tip of the scanner was rotated by 45º, and first the buccal surface then the palatal surface was scanned. Each intraoral scanner device made eight digital impressions, resulting in 8 STL files for the comparison procedure under standard lighting conditions. STL files were imported into the Geomagic Control X program and were compared to the reference model by superimposition. In Geomagic Control X, all STL files were cropped by the same examiner to eliminate any unnecessary parts such as the palatal soft tissue and tuber maxillae. First, an initial alignment was performed, which matched the coordinate system of scan data to the nominal data; then, the best-fit alignment function was used to find the best overall alignment. After superimposition, 3D comparison (gap distance assessment of available surface points) between the reference STL files and files produced by the tested intraoral scanner devices was performed. The measured parameters were the following (Fig. 2):

- Surface deviation of the full-arch scan: Variation between the digital impressions made by the examined IOSs and the reference dataset. It represents the global accuracy of the full arch. (Parameter 1).
- Surface deviation of the abutment teeth 14 and 17 and the alveolar ridge between them (four-unit bridge): It presents the distortion effect of the edentulous ridge. (Parameter 2).
- Surface deviation of the prepared incisor [11]: this parameter indicates a single tooth impression capability – the best accuracy of the IOS (Parameter 3).

The 3D analysis software exported the root mean square (RMS) values of spatial deviations for use in a statistical software package. The arithmetic means of the RMS values were used to indicate the trueness, and their standard deviation (SD) parameters were used to indicate the precision of the investigated scanner based on an article by Renne, Mennito and Vág in 2021 [30].

Eight individual RMS data points were available for each of the three Parameters described above and each IOS, resulting in a dataset of 96 observations. Scanner performance was described in terms of mean ± SD of RMS. Device versions were compared pairwise using Student's two-sample t-test (if normality assumptions were satisfied) or Wilcoxon's rank-sum test (otherwise). The statistical package Stata was used for data handling and analysis.

## Results

**Fig. 3:**
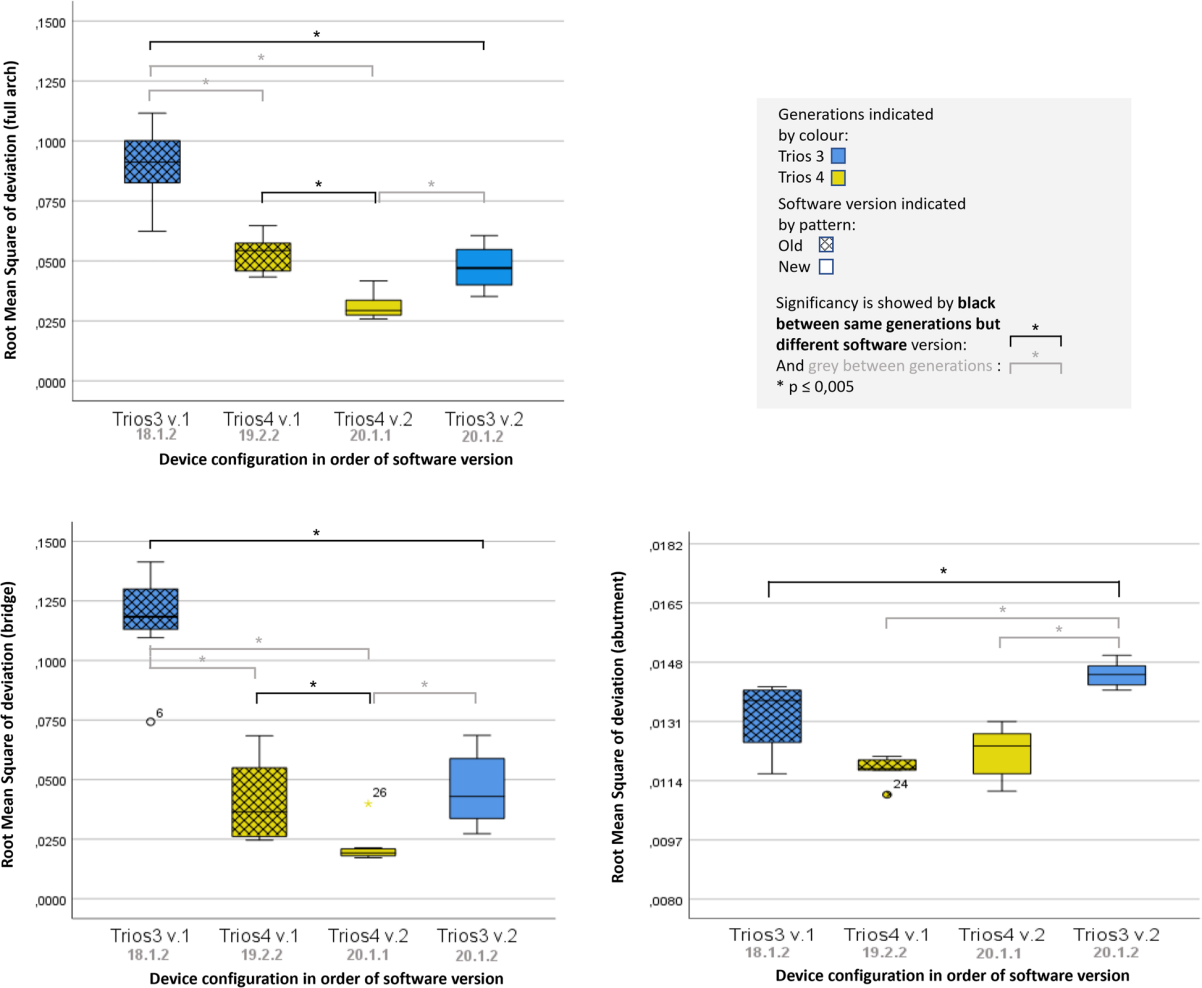
Boxplot diagrams of the results. The intraoral scanners are ordered according to the software release date

Significant differences were found between the generations and between the software versions. The differences in tendencies were not linear, so we cannot state that newer generations and newer software always reproduce the geometry of the oral cavity in a more accurate fashion. The final result depends also on the region under investigation (Table 2, Fig. 3).

**Table 2:** Table of results (μm)

	**Scanner**	**Mean**	**SD**	**Min**	**Max**	**iqr**
**Parameter 1**	*Trios3 v.1*	90,24	15,35	62,40	111,60	17,55
	*Trios3 v.2*	47,44	9,17	35,20	60,60	14,80
	*Trios4 v.1*	52,91	7,44	43,30	64,70	11,60
	*Trios4 v.2*	31,06	5,24	25,90	41,70	6,20
**Parameter 2**	*Trios3 v.1*	117,35	20,11	74,30	141,40	16,85
	*Trios3 v.2*	45,86	14,84	27,30	68,60	25,15
	*Trios4 v.1*	41,04	16,48	24,60	68,40	28,85
	*Trios4 v.2*	21,69	7,50	17,20	39,90	2,45
**Parameter 3**	*Trios3 v.1*	13,26	0,94	11,60	14,10	1,50
	*Trios3 v.2*	14,45	0,36	14,00	15,00	0,55
	*Trios4 v.1*	11,75	0,35	11,00	12,10	0,30
	*Trios4 v.2*	12,21	0,71	11,10	13,10	1,15

### Accuracy results of parameter 1 (average surface deviation across a full-arch digital impression)

In the case of full-arch digital impressions the accuracy results from lowest to highest accuracy are the following: TRI3_1 (90.24 ± 15.35 µm), TRI4_1 (52.91 ± 7.44 µm), TRI3_2 (47.44 ± 9.17 µm), and TRI4_2 (31.06 ± 5.24 µm). The newest software version with the older hardware cannot reach the accuracy level of the newer hardware with the second-latest software. The updated software reached significantly better accuracy in both generations. We did not find a significant difference in accuracy between TRI4_1 and TRI3_2, which suggests that with the proper software update, the older generation could perform similarly to new equipment in terms of accuracy.

### Accuracy results of parameter 2 (average surface deviation across a four-unit bridge)

The observed data showed a decrease in the deviation from perfect trueness and precision as hardware and software evolved.

The accuracy results of teeth 14 and 17 and the edentulous ridge in decreasing order of deviation were the following: TRI3_1 (117.35 ± 20.11 µm), TRI3_2 (45.86 ± 14.84 µm), TRI4_1 (41.04 ± 16.48 µm), and TRI4_2 (21.69 ± 7.50 µm). We found statistically significant differences between the hardware generations and between the majority of software versions, indicating that the newer versions could create more accurate digital impressions, and the distortion effect of the alveolar ridge is getting reduced – the stitching mechanism of the updated software algorithm seems to work better. Although TRI3_2 and TRI4_1 did not have significant accuracy differences between them it can be seen again that the old generation with newer software can reach the accuracy level of the new IOS models.

### Accuracy results of parameter 3 (surface of the prepared incisor – 11)

The accuracy results of the prepared tooth 11 in decreasing order of deviation were the following: TRI3_2 (14.45 ± 0.36 µm), TRI3_1 (13.26 ± 0.94 µm), TRI4_2 (12.21 ± 0.71 µm) and TRI4_1 (11.75 ± 0.35 µm). We found a significant difference between TRI3_1 and TRI3_2, but no significance was shown between TRI4_1 and TRI4_2. With this parameter, the latest software produced lower accuracy. The lack-of-precision assessment of the TRI4_2 also produced a greater value. All deviations from perfect trueness were below 20 µm, which is comfortably within the clinically acceptable range.

## Discussion

Numerous studies investigated the different properties of IOS: scanning speed, ergonomic properties, special features, accuracy [8, 25, 34–37]. In this in vitro study the accuracy of intraoral scanners were measured. Accuracy can be measured with different methods. Most studies use special software to compare STL files to a reference data set [7]. The most widely used method is the best-fit alignment. Another possibility is linear distance measurement when gaps between pairs of points are measured [19, 38, 39]. Accuracy has been assessed in many cases by examining prosthetic workflows [7, 40, 41].

In our present study, best-fit alignment was performed. Two different generations of intraoral scanners were used: 3Shape Trios 3 and 3Shape Trios 4. A generation change means, that the manufacturing company upgrades an already existing intraoral scanner (previous generation) in terms of both hardware and software, creating a new version (new generation model) [35]. A software update means a series of changes to fix or improve the program run by the computer. In the literature, limited information is available about the impact of software updates on intraoral scanners performance [30].

This study investigated the effect of software updates on the accuracy of IOS devices. Two different generations of 3Shape Trios intraoral scanners (Trios 3 and Trios 4) and four different software versions were evaluated for their accuracy (trueness and precision). For accuracy, in the literature, the clinically acceptable range for the marginal fit of a restoration is 50 to 120 μm [9, 10]. Examining the accuracy of the full arch is essential because situations when a longer-span or full-house bridge needs to be fabricated occur commonly. Investigating the accuracy of the deviation between two abutments is relevant because of the edentulous region, which can affect the accuracy. The accuracy of a prepared tooth 11 for a crown is vital for examining chairside systems when the indication is a short-span fixed dental prothesis (FPD).

The null hypothesis was rejected on analysis of full arch and bridge scan data: an association between the recency of the software version of the IOSs and a greater accuracy of digital impressions was found.

In case of parameter 1, there was a significant improvement in trueness and precision. The Trios 3, with the latest software version (TRI3_2) was more accurate than the Trios 4 Move with the previous software version (TRI4_1), which is the latest generation of the 3Shape company’s IOS devices. Furthermore, the accuracy of the software updated Trios 4 Pod intraoral scanner (TRI4_2) was better than that of the mentioned Trios 3 (TRI3_2). The latest generation of 3Shape products (Trios 4 Pod) with the updated software (TRI4_2) produced the best accuracy result in our study, except in case of parameter 3, where the Trios 4 Pod with the older software (TRI4_1) proved to have the highest trueness. It is important to highlight that the software run by the Trios 4 Pod was not the latest software version in the dental market. Based on the result, it could be hypothesised that the hardware version of the IOS could impact the accuracy. Ender et. al. [22] in 2019 investigated the full arch and posterior region with two software versions of the CEREC Omnicam. The updated software version produced better accuracy in both parameters.

In the case of parameter 2 significant differences could be seen between the results of the oldest (TRI3_1) and the latest (TRI3_2) software versions of Trios 3 intraoral scanners. The measured data of parameter 2 showed an increasing tendency in trueness and precision compared to parameter 3 (surface deviation of the prepared incisor). It has been stated in previous studies that longer captured distances result in less accurate digital impressions [13].

An edentulous ridge between teeth 14 and 17 is an important part of our measurement because most scanners capture the surfaces with increased inaccuracy across edentulous ridges. Edentulous regions contain less information than tooth surfaces [14, 42]; therefore, it is more difficult for the IOS to stitch the images together. In addition, the size of the edentulous region can affect the recognition of the overlaps, thereby the accuracy [38]. Kim et al. [43] in 2017 investigated the accuracy of IOS on the edentulous region using a 3Shape Trios 3 intraoral scanner device. The average accuracy of the examined IOS (3Shape Trios 3) was 36.1 ± 13.0 µm. In our research, Trios 4 with software version 19.2.2. (TRI4_1) produced almost the same accuracy (41.04 ± 16.48 µm). Furthermore, the Trios 4 with updated software (TRI4_2) provided better accuracy results in the case of trueness and precision (21.69 ± 7.50 µm). The examined edentulous space in our study was shorter than in the mentioned article, which may have caused differences between the results.

In the case of parameter 3, negative impact of software updates on trueness was found. The range of variation across scanner configurations was less than 5 µm, so the difference's clinical impact is negligible. In our research more accurate data were obtained than in the literature: Park et al. [44] examined the accuracy of the 3Shape Trios 3 intraoral scanner in 2016. The accuracy of the examined parameter (solo crown – first incisor) was 49.7 ± 13.0 µm, which is less accurate than our results: TRI3_2, 14.45 ± 0.36 µm and TRI3_1, 13.26 ± 0.94 µm. In our research, two software versions were evaluated in Trios 3: 18.1.2. and 20.1.2. Version 18.1.2. was released in 2018 and 20.1.2. was introduced to the dental market in 2020. The previous research by Park et al. was conducted in 2016. Accordingly, their Trios 3 intraoral scanner was running an earlier software version, which may explain the differences between the results. Another research by Zimmerman et al. [45] examined the accuracy of prepared teeth for solo restorations using the CEREC Omnicam with two different software versions (version 4.6.1 and version 5.0.0). The results showed no significant difference between the two versions in accuracy (v. 4.6.1: 36.7 µm; v. 5.0.0: 40.5 µm). Based on our results, IOSs with legacy software produced almost the same results as IOSs running updated software versions. The difference from the data in the literature may also be due to the shape of the prepared abutment, the distance from the neighbouring teeth as they affect the IOS’s field of vision and thus its accuracy. All measured object has a different geometry making the results not completely comparable [46, 47].

Few studies in the literature evaluate the effect of software updates on the accuracy of intraoral scanners [30]. In further studies, it would be necessary to describe the software version of the IOS and not only the hardware type.

There are some limitations to this study. Our results are based on model scanning procedures (in vitro study); it could be interesting to measure these parameters in clinical circumstances. In clinical situations, numerous factors can impact the accuracy of IOS, such as the saliva flow rate in the oral cavity, the individual characteristics of the patient, and the operator's skills in digital impression-taking. Moreover, our study used only two scanners from the same manufacturer. They use confocal laser technology, and the method of recording data is a video sequence [48]. Many types of intraoral scanners with different data capture modes can produce different results. It would also be interesting to investigate additional intraoral devices for the effects of software updates.

## Conclusion

Based on our results the null hypothesis was rejected. Within the current study's limitations, we conclude that new generations of intraoral scanner hardware and software can significantly increase the trueness and precision of the devices when it comes to full-arch scanning. The presence of an edentulous region may still adversely affect the accuracy of the results; however, software updates seem to achieve more accurate STL files. All versions create a clinically acceptable digital impression of a single abutment.

## Data Availability

The datasets used and/or analyzed in the current study are available from the corresponding author upon reasonable request.
